# Numerical simulations of an integrated radio-frequency/wireless coil design for simultaneous acquisition and wireless transfer of magnetic resonance imaging data

**DOI:** 10.1088/1361-6560/acd614

**Published:** 2023-06-08

**Authors:** Devon K Overson, Julia Bresticker, Devin Willey, Fraser Robb, Allen W Song, Trong-Kha Truong, Dean Darnell

**Affiliations:** 1 Brain Imaging and Analysis Center, Duke University, Durham, NC, United States of America; 2 Medical Physics Graduate Program, Duke University, Durham, NC, United States of America; 3 University of Virginia, Charlottesville, VA, United States of America; 4 GE Healthcare, Inc, Aurora, OH, United States of America

**Keywords:** MRI, imagning, wireless, WIFI, magnetic resonance imaging, simulations

## Abstract

*Objective.* A novel magnetic resonance imaging (MRI) radio-frequency (RF) coil design, termed an integrated RF/wireless (iRFW) coil design, can simultaneously perform MRI signal reception and far-field wireless data transfer with the same coil conductors between the coil in the scanner bore and an access point (AP) on the scanner room wall. The objective of this work is to optimize the design inside the scanner bore to provide a link budget between the coil and the AP for the wireless transmission of MRI data. *Approach.* Electromagnetic simulations were performed at the Larmor frequency of a 3T scanner and in a WiFi wireless communication band to optimize the radius and position of an iRFW coil located near the head of a human model inside the scanner bore, which were validated by performing both imaging and wireless experiments. *Main Results.* The simulated iRFW coil with a 40 mm radius positioned near the model forehead provided: a signal-to-noise ratio (SNR) comparable to that of a traditional RF coil with the same radius and position, a power absorbed by the human model within regulatory limits, and a gain pattern in the scanner bore resulting in a link budget of 51.1 dB between the coil and an AP located behind the scanner 3 m from the isocenter, which would be sufficient to wirelessly transfer MRI data acquired with a 16-channel coil array. The SNR, gain pattern, and link budget for initial simulations were validated by experimental measurements in an MRI scanner and anechoic chamber to provide confidence in this methodology. These results show that the iRFW coil design must be optimized within the scanner bore for the wireless transfer of MRI data. *Significance.* The MRI RF coil array coaxial cable assembly connected to the scanner increases patient setup time, can present a serious burn risk to patients and is an obstacle to the development of the next generation of lightweight, flexible or wearable coil arrays that provide an improved coil sensitivity for imaging. Significantly, the RF coaxial cables and corresponding receive chain electronics can be removed from within the scanner by integrating the iRFW coil design into an array for the wireless transmission of MRI data outside of the bore.

## 1. Introduction

In magnetic resonance imaging (MRI), data transfer between the scanner hardware components and the console computer outside of the scanner room is central to the scanner’s operation. For example, image data acquired with radio-frequency (RF) coil arrays, which have increasingly large numbers of coil elements (Schmitt *et al* 2008, Wiggins *et al* 2009), is transferred over an elaborate network of wired connections to the console computer for image reconstruction. All of these wired connections require complex cable routing inside the scanner with expensive RF cables, filters, and baluns to maintain the MR image quality (Peterson *et al* 2003, Fujita *et al* 2013). These cable assemblies can add significant weight and bulk to the coil array, which increase patient setup time and may cause patient discomfort, and they must be carefully placed around the patient to avoid burn risks (Dempsey *et al* 2001, Ganti *et al* 2021). To address these limitations, a previous study has proposed to split a typical RF coil array cable assembly into two separate cable assemblies and to use inductively-coupled RF ‘sniffer’ coils to transfer the image data between them (Bulumulla *et al* 2015). This design is more technologist-friendly because only one short cable assembly is connected to the coil array while the other cable assembly is embedded in the scanner table. However, it requires time-consuming, precise alignment of the ‘sniffer’ coils and has been shown to degrade image quality. Alternatively, another study has proposed to replace the cable assembly between the RF coil array and the scanner with a wireless data transfer system that uses two separate millimeter-wave dipole antenna arrays placed close to one another within the scanner bore to transfer large image data sets (Aggarwal *et al* 2017). However, this system requires costly additional electronics (e.g. amplifiers, signal dividers, fiber optic cables) and significant scanner modifications, which is impractical for existing MRI scanners.

We recently proposed a novel integrated RF/wireless (iRFW) coil design that can perform simultaneous MRI signal reception and wireless data transfer between the coil and an access point (AP) located on the scanner room wall that is connected to a computer in the console room (Darnell *et al* 2019). Uniquely, this coil design uses the same coil conductors to: receive the near-field MRI RF signal from a subject in the same way as a traditional RF coil; and perform wireless data transfer by transmitting (or receiving) a radiated signal to (or from) the AP, without requiring any scanner modifications or additional antenna systems in the scanner bore. In fact, the iRFW coil’s wireless design reduces the system components in the scanner because the receive chain can be, for example, moved outside of the scanner and into a small enclosure located in the machine room, which can reduce scanner cost, complexity, and increase space in the bore for patients.

In previous proof-of-concept experiments, this coil design was used to acquire MR images with multiple pulse sequences and to wirelessly transfer non-image data at a low data rate (LDR) of ∼11 Mbps between the coil and the AP, with no degradation in the image or wireless data quality (Darnell *et al* 2019). It has further been used for LDR applications such as wireless localized *B*_0_ shimming of the spinal cord (Cuthbertson *et al* 2022) and brain (Cuthbertson *et al* 2020) with integrated RF/shim coil arrays, wireless respiratory monitoring with a respiratory belt (Cuthbertson *et al* 2020), wireless Q-spoiling (Cuthbertson *et al* 2021), and wireless physiological motion monitoring with an ultrasound sensor (Willey *et al* 2022).

However, the wireless transfer of MRI data requires a higher data rate, which has been estimated to be ∼8 Mbps/coil element (Nohava *et al* 2020, Darnell *et al* 2022) or ∼128 Mbps for a 16-channel clinical coil array. For this high data rate (HDR) wireless data transmission, the transceiver modules of both the AP and iRFW coil can have a significantly lower receiver sensitivity (i.e. higher noise floor) than for LDR applications. Clearly, the amount of radiated signal transmitted from the iRFW coil and received by the AP (or conversely) must be above the receiver sensitivity of the HDR transceiver used by the coil and the AP to avoid any data loss. In contrast to previous wireless RF coil designs (Aggarwal *et al* 2017) and our previous iRFW coil designs for LDR applications, the iRFW coil design for HDR applications needs to be optimized in the scanner bore as part of the entire MRI system to ensure that the radiated signal emitted from the coil to the AP is above the receiver sensitivity. In this work, we therefore perform new simulations and experiments to determine the iRFW coil design within the scanner bore that maximizes the radiated wireless signal transmitted between the coil and the AP, while still providing the same MRI signal-to-noise ratio (SNR) and subject specific absorption rate (SAR) as those of a traditional RF coil design. Initial simulations have been previously presented in abstract form (Bresticker *et al* 2019).

## 2. Theory

### 2.1. Coil and system design

**Figure 1.**
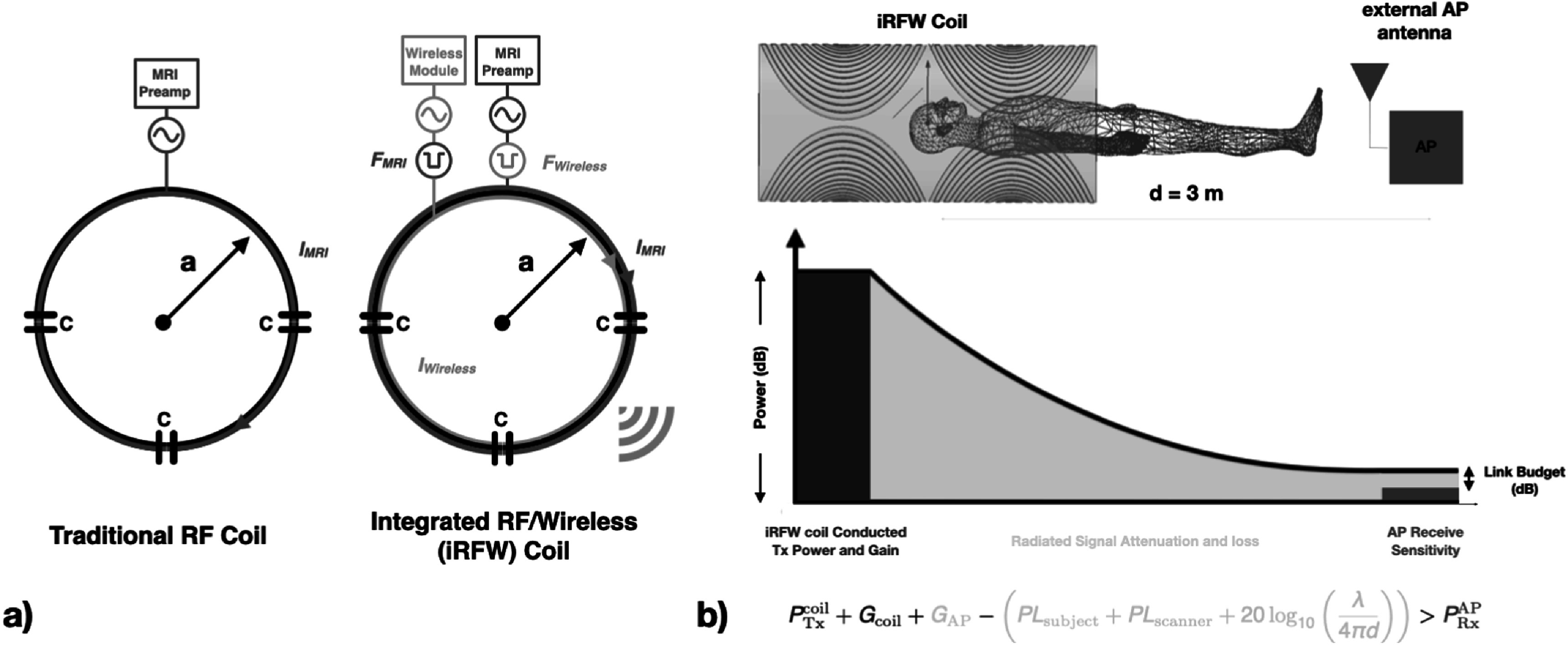
An iRFW coil (a) uses the same coil conductors for RF signal reception and wireless data transfer between the coil in the scanner bore and an AP placed on the scanner room wall. The quality of the wireless data transfer between the coil in the scanner bore and the AP is determined by the wireless module’s conducted transmit power and coil gain (green region), the path loss between the coil and the AP gain (beige region), and the receive sensitivity of the AP (red region). This is expressed as the link budget power inequality (b).

The iRFW coil design allows RF currents at the Larmor frequency, *I*_MRI_, and within a wireless communication band, *I*_wireless_, to flow on the same coil conductors for MRI RF signal reception and wireless data transfer (Darnell *et al* 2019), respectively (figure 1(a)). For imaging, the SNR and SAR of the iRFW coil design are made similar to those of a traditional RF coil design by inserting a high-impedance bandstop filter at the Larmor frequency, *F*_MRI_, between the coil conductors and a wireless communication transceiver module (figure 1(a), red bandstop filter), which allows *I*_MRI_ to flow on the conductors for MRI signal reception rather than into to the 50 Ω termination of the module. Similarly, a separate high-impedance bandstop filter at the wireless communication frequency band, *F*_wireless_, is inserted between the coil conductors and the MRI preamplifier (figure 1(a), orange bandstop filter), which allows *I*_wireless_ to flow on the conductors to generate the far-field radiated signal for wireless data transmission (figure 1(a)) rather than into the 50 Ω termination of the preamplifier.

An electrically large iRFW coil (i.e. circumference ∼ wavelength *λ*) can efficiently radiate a signal with no radial field components (i.e. far-field) at its operational frequency when its radius is small relative to its distance to the AP (figure 1(b), *d* ≫ *a*). Specifically, the electromagnetic fields generated by the RF currents flowing on the iRFW coil produce electric and magnetic fields in the radial (*E*_*r*_, *H*_*r*_), polar (*E*_*θ*_, *H*_*θ*_), and azimuthal (*E*_*ϕ*_, *H*_*ϕ*_) coordinate directions relative to the coil center. Far from the coil at the AP, *E*_*r*_ and *H*_*r*_ are diminished by $\sim \tfrac{1}{r}$ so that the only fields that contribute to the radiated transverse electromagnetic wave at the AP are *E*_*ϕ*_ and *H*_*θ*_. As such, the corresponding far-field time-averaged radiated signal intensity is only dependent on the angular distributions of the signal (Stutzman and Thiele 1998, Balanis 2005). For example, an iRFW coil with a radius *a* = 35 mm and RF currents with frequencies of 127.7 MHz (i.e. 3T MRI RF current) and 2.4 GHz (i.e. WiFi RF current) flowing on its conductors will generate a far-field radiated signal at the AP placed 3 m away because the coil radius is small compared to the distance between them. Here, the WiFi RF current will produce a strong radiated signal because its wavelength (12.2 cm) is similar to the coil circumference (22 cm), but the MRI current will have little signal at the AP because its wavelength (2.35 m) is much larger.

The radiated signal from the iRFW coil in the scanner bore is fully characterized by its gain pattern, *G*_coil_(*r*, *θ*, *ϕ*), and corresponding peak antenna gain, ${G}_{\mathrm{coil}}^{\mathrm{peak}}$, which is determined by (1) the ratio of the coil radius to the operational wavelength (Beekman 1986, Burnside *et al* 1987, Balanis 2005) and (2) its operational environment (e.g. MRI scanner). For example, an iRFW coil with a small radius (*a* < *λ*_wireless_) has a free-space gain pattern in an open environment that is isotropically distributed around the circumference of the coil. As the coil radius increases, its radiated signal directed (i.e. antenna directivity) along the normal of the coil also increases, while the signal intensity decreases in the plane of the coil. Further, the gain pattern can become significantly less isotropic when the coil is placed in the scanner bore because the radiated electromagnetic fields are attenuated and/or reflected by the subject and scanner, which changes the amount of radiated signal leaving the scanner bore and received by the AP. As such, the iRFW coil design process must consider both the physical and electrical properties of the coil itself and the MRI scanner environment to maximize the radiated signal for wireless data transfer. Additionally, the iRFW coil design must also provide a local SAR_MRI_ in the subject that is comparable to that of a traditional RF coil and when summed with a SAR_wireless_ operating with a worst-case 100% transmit power duty cycle provide a SAR_total_ (=SAR_MRI_ + SAR_wireless_) that is less than 1.6 W kg^–1^ averaged over one gram of tissue and 2.0 W kg^–1^ averaged over ten grams of tissue (Murphy and Williams 2004, Wang *et al* 2007, Khan *et al* 2015), as required by the Federal Communication Commission (FCC) guidelines found in 47 CFR Part 2, section 2.1093 and in the European Union (EU) IEC 62232, respectively.

### 2.2. Wireless data transmission

The quality of the wireless data transferred between the iRFW coil and the AP is determined by the total amount of radiated signal that is emitted from the coil and received by the AP, which can be quantified by adding the power gains and losses between them (figure 1(b)). Specifically, the wireless data quality is maintained in the scanner if the sum of:

• the maximum transmit power from the wireless transceiver, ${P}_{\mathrm{Tx}}^{\mathrm{coil}}$,
• the maximum radiated signal power, or antenna gain, from the coil, *G*_coil_, and from the AP, *G*_AP_,
• the path losses from the attenuation and/or reflections by the subject, PL_subject_, and by the scanner, PL_scanner_,
• the wavelength-dependent free-space signal path loss between the coil and the AP (separated by a distance *d*), which is independent of the scanner bore,

is greater than the minimum receive power sensitivity (i.e. noise floor) of the AP, ${P}_{\mathrm{Rx}}^{\mathrm{AP}}$. Mathematically, this power inequality can be expressed as (Bose and Foh 2007, Phillips *et al* 2013):

\begin{eqnarray*}{P}_{\mathrm{Tx}}^{\mathrm{coil}}+{G}_{\mathrm{coil}}+{G}_{\mathrm{AP}}-\left({{\mathrm{PL}}}_{\mathrm{subject}}+{{\mathrm{PL}}}_{\mathrm{scanner}}+20{\mathrm{log}}_{10}\left(\displaystyle \frac{\lambda }{4\pi d}\right)\right)> {P}_{\mathrm{Rx}}^{\mathrm{AP}}\end{eqnarray*}

which must hold to prevent wireless data loss. Here, the total path loss, PL, is the sum of PL_subject_, PL_scanner_, and the free-space signal path loss in equation (1). The difference between the summed power terms on the left side and the receiver sensitivity on the right side of the inequality is referred to as the link budget, which is used to evaluate wireless systems.

As previously mentioned, the receiver sensitivity ${P}_{\mathrm{Rx}}^{\mathrm{AP}}$ for HDR wireless data transfer is much higher than for LDR applications, which results in a significantly decreased link budget between the iRFW coil and the AP. As a result, the power terms on the left side of the inequality must be optimized to avoid data loss between the devices. Unfortunately, the coil’s maximum transmit power ${P}_{\mathrm{Tx}}^{\mathrm{coil}}$ in the link budget is limited for each individual modulation scheme by FCC regulations for wireless transmitters and cannot be increased to improve the link budget in the scanner, which leaves only the gain and path loss terms to be optimized for data transmission. The *G*_AP_ can be initially optimized to decrease the path loss by connecting the AP to a highly directive external antenna mounted on the scanner room wall with a peak gain along the axis of the bore, which will couple more radiated signal between the iRFW coil and the antenna. However, the external AP antenna design can in practice only be optimized once and will therefore be fixed for all iRFW coils to be used in the scanner. As such, the *G*_coil_(*r*, *θ*, *ϕ*) of the iRFW coil design must be optimized during the coil design process to provide an improved link budget by determining a coil radius and position in the scanner bore that both minimizes the path loss and maximizes the radiated power coupled between the coil and the AP antenna. Further, the coil must also provide a total local SAR_total_ that is within FCC and EU requirements while providing the same SNR and local SAR_MRI_ as those of a traditional RF coil.

## 3. Methods

### 3.1. MRI system and iRFW coil model

In these simulations, an iRFW coil was modeled (ANSYS HFSS 2019 R3, Canonsburg, PA) in a scanner bore and located near the head of a 182 cm human model made of saline to determine the SNR, SAR_MRI_, SAR_wireless_, and gain pattern of the coil design in a wireless communication band. In this work, the Larmor and wireless communication band frequencies chosen for the simulations were 127.7 MHz and 2.442 GHz, which are used for 3T MRI and WiFi, respectively. First, the coil was modeled by inserting three lumped port capacitors (*C*_1_ = *C*_2_ = *C*_3_ = 10 pF) about the coil perimeter and an embedded RF source composed of two 0.8 × 0.7 mm 50 Ω 1 W RF lumped port single-mode excitations (i.e. MRI and WiFi ports), which generated *I*_MRI_ and *I*_WiFi_ on the coil (Fan *et al* 2015). The ports were RF-isolated from each other at the Larmor and WiFi frequencies by inserting two high-impedance filters, *F*_WiFi_ (${S}_{21}^{\mathrm{WiFi}}$ = −36.9 dB) and *F*_MRI_ (${S}_{21}^{\mathrm{MR}}$= −25.4 dB), between the coil and their matching circuits, *M*_MRI_ and *M*_WiFi_, which could be adjusted to ensure that the coil response was in tune for each simulation.

**Figure 2.**
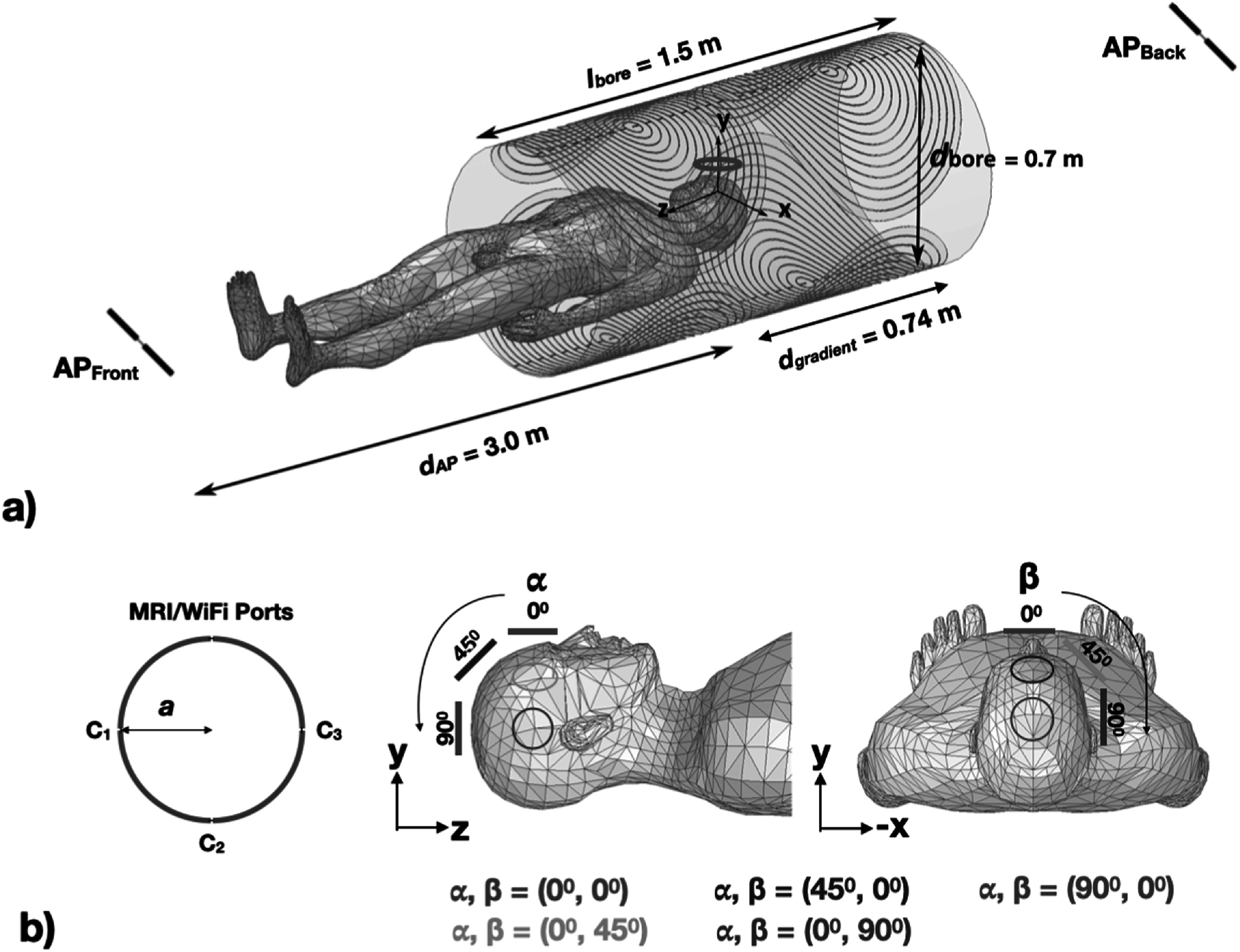
The MRI scanner bore was modeled as a cylindrical two-layer impedance boundary surrounding a human model with two dipole antennas (AP_back_ and AP_front_) placed 3 m from the scanner isocenter. The gradient coils have diameters between 4.6 and 74 cm with a 0.2 cm spacing between adjacent gradient coil elements (a). Simulations were performed with the iRFW coil placed in the model at five separate locations about the head for 8 different coil radii (b).

Next, the MRI scanner bore (${l}_{\mathrm{bore}}$= 1.5 m, ${d}_{\mathrm{bore}}$ = 70 cm) based upon a GE MR750 MRI scanner was modeled as an infinitesimally thin cylindrical sheet with a 2-layer impedance boundary condition assigned to have a 2 mm copper layer (WiFi skin depth ≈1.2 *μ*m Wolff and Dellinger 1910) and 50 mm FR4-epoxy layer (loss tangent ≈0.02 at the WiFi frequency Geyer *et al* 2005) on its surface. This model is electrically equivalent to a 52 mm thick cylindrical bore model of the same materials but drastically reduces the computational cost of the simulations. Further, a 50 Ω terminated gradient coil with 64 turns was modeled on the scanner bore (figure 2(a)) to simulate the absorbed and reflected WiFi radiated signal interactions within the scanner bore. Additionally, two APs, AP_front_ and AP_back_, were modeled as 2.4 GHz WiFi ideal half-wavelength dipoles placed 3m away from the bore isocenter along the *z*-axis in the superior-inferior direction. The APs were assumed to be ideal dipole antennas, which are often used in commercially available access points (e.g. Airport Extreme, Apple Inc.) for wireless data transfer that are readily available to install in an MRI scanner room. Finally, a single radiation boundary was added around all these objects to absorb any additional radiated power that leaves the scanner bore, but is not received by the APs, to ensure that it does not contribute to the link budget.

### 3.2. Simulations

Electromagnetic (EM) simulations of the iRFW coil in the scanner were performed for eight coil radii (*a* = 15, 20, 25, 30, 35, 40, 45, and 50 mm) each at five different positions (*α* = 0°, 45°, 90° and *β* = 45°, 90°) (figure 2(b)) for a total of 5 × 8 = 40 unique simulations. For each simulation, the average SNR, SNR coefficient of variation (COV; defined as the standard deviation divided by the mean), and maximum SNR were calculated in: the entire head; a −3 dB region-of-interest (ROI) relative to the maximum SNR; and a −10 dB ROI relative to the same maximum SNR. The simulations were used to determine the iRFW coil size and position that provided: (1) a comparable average SNR, SNR COV, maximum SNR, and SAR_MRI_ to those of a traditional RF coil; (2) a low local SAR_WiFi_ deposited into the head; and (3) a *G*_coil_ resulting in the largest link budget between the coil and the APs.

First, the SNR distribution for each coil radius–position combination was calculated on the meshed model by dividing the magnetic field in the human model by the resistance of the coil and of the human model (Roemer *et al* 1990). Here, radiated losses have been excluded from the SNR calculations because it has been shown that 99.98% of the input power delivered to a traditional RF coil is dissipated into the subject for simulation models that include a metal scanner bore object and RF screen room radiation boundary (Collins *et al* 2003). Next, the SAR_MRI_ and SAR_WiFi_ distributions were determined for the same combinations and the 1 g and 10 g averaged SAR values were calculated in the head model by finding the peak SAR and integrating the neighboring SAR values in volumes corresponding to 1 g and 10 g of tissue, respectively, for both the Larmor and WiFi frequencies. The SAR_MRI_ and SAR_WiFi_ were then summed to determine the SAR_total_ (= SAR_MRI_ + SAR_WiFi_) to compare with both the FCC and EU regulations (Ketterl *et al* 2013, Arduino *et al* 2018, Chiaramello *et al* 2019, Golestanirad *et al* 2019). The corresponding wireless gain patterns and the *S*_21_ values between the coil and the APs were recorded to determine the coil radius–position combination that provided the best path loss for wireless data transfer in the scanner bore. Finally, the same SNR and SAR_MRI_ simulations were performed for a traditional RF coil with the same coil radii and positions for comparison with the iRFW coil.

A separate set of simulations were then performed with the best iRFW coil radius–position combination for nine different human model heights that were equally spaced between 168 and 194 cm in the *z*-direction to determine the impact of body size on the path loss, and therefore the link budget, between the coil and the APs. Here, the coil’s terminal impedance for the WiFi port was not adjusted because the distance between the coil and the human model remained fixed for all heights. Lastly, simulations for an iRFW coil with radius *a* = 35 mm were performed without the human model, scanner bore, gradient coil, or APs: (1) on a rectangular water phantom to determine its SNR and (2) with no water phantom to determine its unattenuated ‘free-space’ gain pattern to compare with experimental measurements.

### 3.3. Simulation workflow

**Figure 3.**
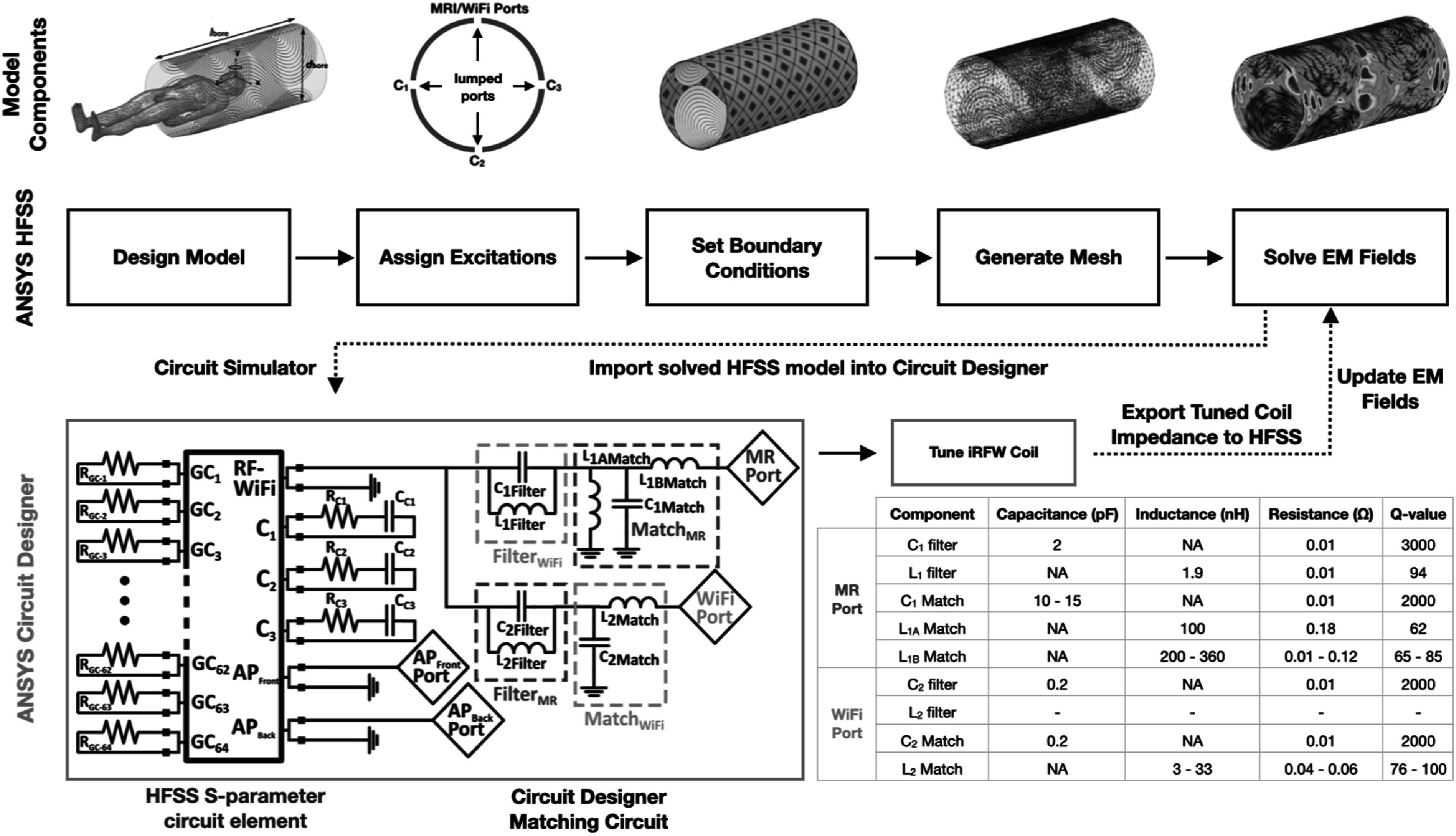
In HFSS, the model is first designed and excitations are assigned to the iRFW coil, gradient coil, and APs. After boundary conditions are assigned, the model is meshed and the initial electromagnetic fields are solved (blue boxes). The object’s terminal impedances are then passed to a circuit designer program (green box) where the MRI and WiFi ports are matched to 50 Ω and the electromagnetic fields are subsequently updated in HFSS. The table shows the (1) electrical characteristics of the components used in the filters and matching circuits (2) and the range of capacitor and inductor values used in the matching circuts for all radius–position combinations. The indcutor ${L}_{2}$ filter was not populated in these simulations and experiments because the large difference between the Larmor and WiFi frequencies allows a single capacitor to be used, which results in a higher *Q*-value of the filter.

The iRFW coil simulations were performed using the embedded ANSYS HFSS electromagnetic simulator and circuit designer programs. The electric and magnetic fields generated by the modeled iRFW coil with the aforementioned lumped port excitations (i.e. capacitors and RF excitations) and assigned boundary conditions were first simulated in HFSS on an automatically generated iterative curvilinear adaptive mesh at the Larmor frequency (i.e. 127.7 MHz) and the 2.4 GHz WiFi center frequency (i.e. 2.442 GHz), which were required to converge within 10 mesh iterations and have less than a 0.02 change in the coil’s return loss for both frequencies (figure 3(a), blue boxes).

Next, the iRFW coil’s MRI and WiFi terminal impedances from the solved HFSS simulations were passed to the circuit designer program schematic via a graphical block diagram element (figure 3(b), green boxes), which allowed the coil response to be tuned for each coil radius and position around the head model. Specifically, the HFSS block diagram element was imported into the circuit and connected to the filters *F*_MRI_ and *F*_WiFi_ followed by the matching circuits *M*_MRI_ and *M*_WiFi_ for each of the MRI (figure 3(b), dashed red boxes) and WiFi (figure 3(b), dashed orange boxes) ports, respectively. The component values for the MRI and WiFi matching circuits were then adjusted in the circuit designer program to ensure that for each coil radius and position, the MRI and WiFi coil responses had an *S*_11_ < − 20 dB, and that the isolation between the two ports was not impacted.

The circuit designer matched impedances were then passed back into the HFSS simulation and applied to the iRFW coil to adjust the corresponding electric and magnetic fields in the model for each of the frequencies. Finally, the impedance mismatch-compensated fields were used to calculate the SNR, SAR_MRI_, SAR_WiFi_, and gain using the embedded ANSYS field calculator program and Matlab (The MathWorks, Natick, MA) for each coil radius, position, and body height simulation. All of these simulations were performed on a shared 56-node Linux Grid Engine cluster with 976 CPU cores and 7.5 TB of RAM.

### 3.4. Experimental measurements

Both a traditional RF coil and an iRFW coil with the same dimensions were constructed to acquire experimental SNR maps for comparison with each other and with the corresponding simulated SNR maps. Additionally, the iRFW coil was also used to acquire experimental free-space WiFi gain patterns for comparison with the corresponding simulations. These comparisons provided confidence to move forward with the remaining simulations including the scanner bore and human model.

Specifically, a traditional RF coil with a typical coil radius (*a* = 35 mm) for MRI applications was first constructed by placing three 10 pF capacitors about the coil perimeter and the coil was tuned to resonate at the 3T Larmor frequency by connecting it to a calibrated 4-port vector network analyzer (VNA) and by adjusting the matching circuit, *M*_MRI_, until the return loss S11 at this frequency was less than –20 dB. MRI experiments were then performed in a 3T Premier Ultra-High Performance MRI scanner (GE Healthcare, Milwaukee, WI) with the coil placed on a rectangular water phantom. An SNR map was acquired using a gradient-echo pulse sequence (repetition time (TR) = 300 ms, echo time (TE) = 1.5 ms, voxel size = 4 × 4 × 4 mm) and was calculated by dividing the signal intensity of each pixel by the standard deviation of the background noise outside the phantom and by multiplying by a non-Gaussian noise correction factor of 0.66 (Kaufman *et al* 1989, Harkin and Ingham 2022).

Next, the traditional RF coil was modified into an iRFW coil by inserting the two custom high-impedance filters, *F*_WiFi_ and *F*_MRI_, between the coil conductors and the original *M*_MRI_ or a new matching circuit, *M*_WiFi_, respectively. The coil’s MRI and WiFi ports were tuned to resonate at the 3T Larmor frequency and in the WiFi frequency band by connecting it to the same 4-port VNA and by adjusting their matching circuits, *M*_MRI_ and *M*_WiFi_, located behind the filters until the return loss S11 at each frequency was less than –20 dB. Further, the S21 was measured to ensure that the isolation between the ports from the filters was less than –25 dB to maintain the RF and wireless performance. Lastly, the *Q*-values of the iRFW coil with (*Q*_loaded_ = 18.9) and without (*Q*_unloaded_ = 19.1) the phantom were measured with no changes to the filters or matching circuit to determine its sensitivity to dielectric loading. For the SNR map, a 50 Ω resistive load was placed behind the *M*_WiFi_ to represent the equivalent WiFi port termination used in the simulations.

**Figure 4.**
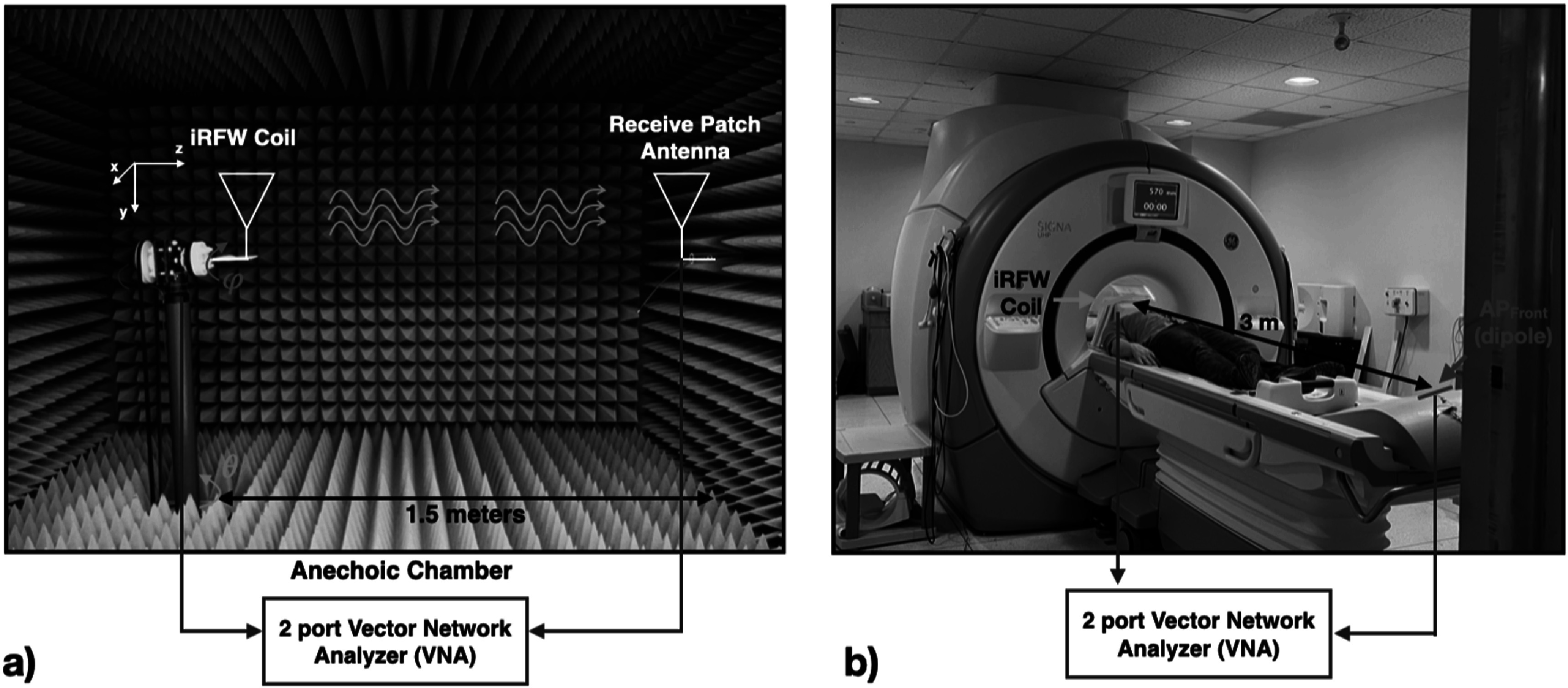
The iRFW coil’s free-space gain pattern was measured in a fully anechoic chamber (a) and *S*_21_ measurements were performed between the iRFW coil placed near a subject’s head in the scanner bore and a 2.4 GHz dipole placed near the scanner room wall for comparison with the simulations.

The iRFW coil’s gain pattern at the WiFi frequency was measured in a 1.5 m calibrated anechoic chamber (figure 4(a)), which eliminated all reflected power in the gain measurement, to determine the free-space gain pattern for comparison with the free-space simulation. Specifically, the gain pattern was generated by performing a set of radiated *S*_21_ measurements between the coil and a patch antenna located inside the anechoic chamber, which were both connected to the VNA, in 5° steps for both the polar (*θ*) and azimuthal (*ϕ*) directions. Next, the coil peak gain was determined by finding the maximum gain recorded from the measurements. As in the MRI experiments, a 50 Ω resistive load was placed behind the *M*_MRI_ to represent the equivalent MRI port termination used in the simulations.

Finally, the simulated path loss between the iRFW coil with the optimal radius and position and the AP_front_ was validated in the scanner bore by measuring the isolation (i.e. *S*_21_) on the VNA between the iRFW coil and a dipole tuned to resonate in the WiFi communication band (figure 4(b)). Specifically, the iRFW coil was placed at the optimal position near the subject’s head with the help of a styrofoam fixture located at the scanner isocenter and the dipole was placed 3 m away from the coil near the scanner room wall. RF baluns were then attached between both the coil and the dipole and their respective coaxial cables connected to the VNA to remove any RF currents on the cable outer conductors, which would otherwise change the isolation measurements. Next, isolation measurements in the WiFi communication band were taken with no subject between the coil and the dipole for comparison with the simulated path loss between the coil and the AP_back_. Additionally, another set of isolation measurements were taken with each of two healthy subjects (heights of 178 and 188 cm) that provided consent laying between the coil and the dipole for comparison with the simulated path loss between the coil and the AP_front_. Next, the average isolation was calculated for each of these three measurements by averaging the data over a 20 MHz bandwidth about the simulated 2.442 GHz WiFi frequency. This experiment was then repeated for the same coil positioned on the styrofoam fixture at the least optimal position determined by the simulations. Lastly, the iRFW coil *Q*-values were measured outside the scanner with (*Q*_loaded_ = 18.9) and without (*Q*_unloaded_ = 18.8) the subjects head inside the sytrofoam fixture in the same way as with the phantom to determine its sensitivity to human subjects.

## 4. Results

### 4.1. Simulations

**Figure 5.**
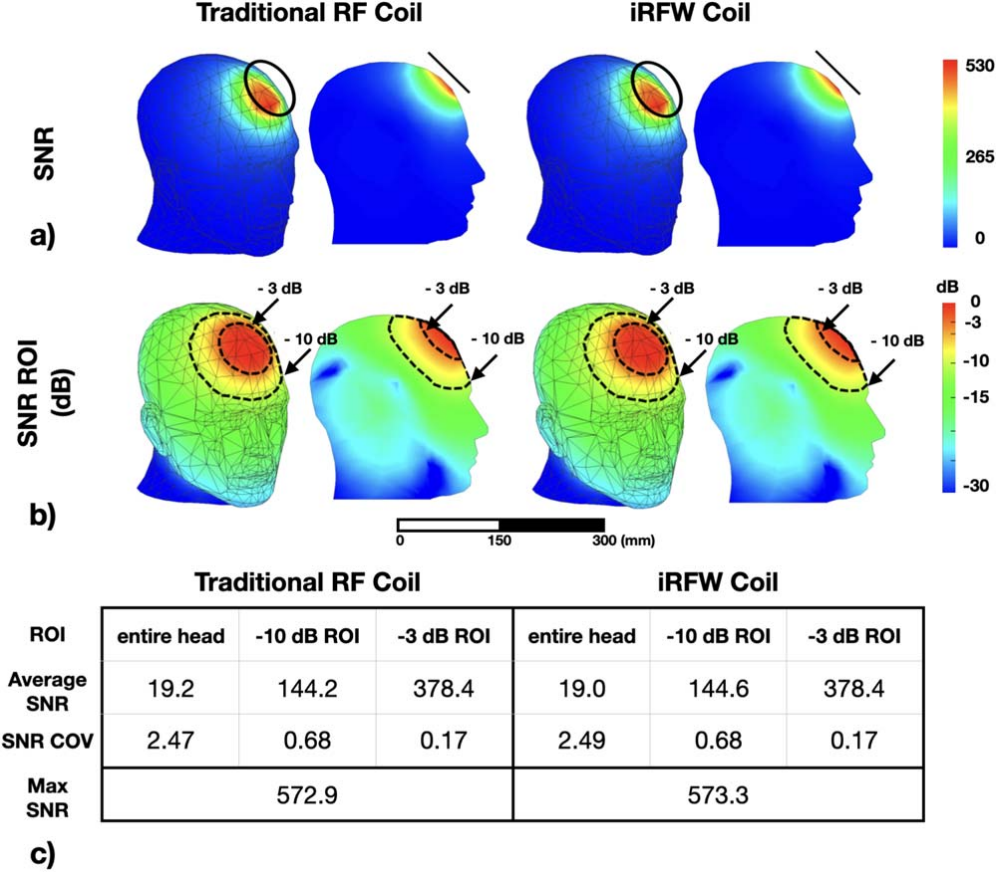
(a) SNR maps for the simulated *a* = 40 mm traditional RF coil and iRFW coil placed at the optimal (*α* = 45°, *β* = 0°) position. (b) Same SNR maps in dB showing the –3 dB ROI and –10 dB ROI. (c) Average, COV, and maximum SNR values in the whole head and in both ROIs, showing that they are virtually identical for both coils.

The optimal iRFW coil radius–position combination among all simulated combinations was found to be (*a* = 40 mm, *α* = 45°, *β* = 0°) based on the aforementioned optimization criteria. The maximum SNR, average SNR, and SNR COV in the entire head were 572.9, 19.0, and 2.49 for the optimal coil radius–position combination (figure 5(a)), respectively, which were nearly the same as those of a traditional RF coil with the same radius and position (figure 5(c)), and they only changed by a maximum of 0.82% for all coil radii and positions. Similarly, the SNR metrics in both the –3 dB and –10 dB ROIs were also in strong agreement for both types of coils and only changed by a maximum of 0.77% for all coil radii and positions.

**Figure 6.**
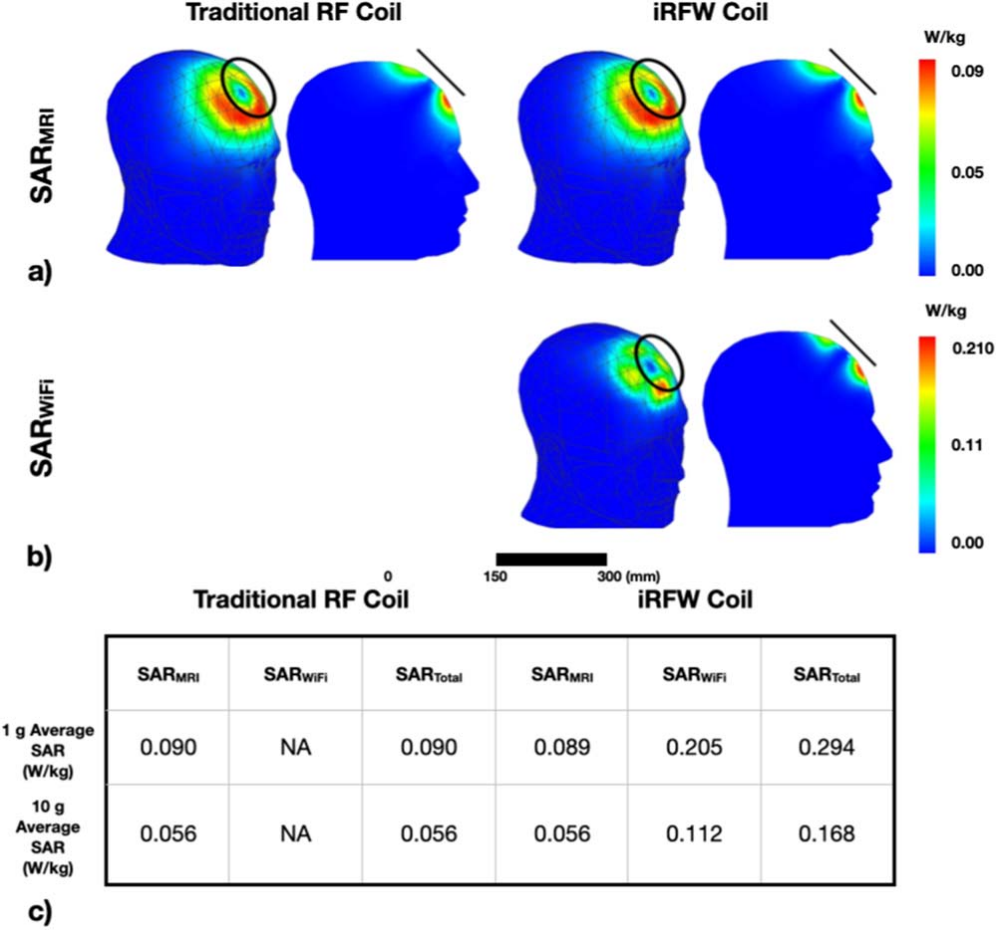
(a) SAR_MRI_ maps for the simulated *a* = 40 mm traditional RF coil and iRFW coil placed at the optimal (*α* = 45°, *β* = 0°) position. (b) SAR_WiFi_ maps for the simulated *a* = 40 mm iRFW coil placed at the optimal (*α* = 45°, *β* = 0°) position. (c) 1 g averaged and 10 g averaged SAR_MRI_, SAR_WiFi_, and SAR_total_ values in the whole head, showing that the SAR_MRI_ values are nearly the same for both types of coils and that the SAR_total_ values for the iRFW coil are well within FCC and EU regulatory limits.

**Figure 7.**
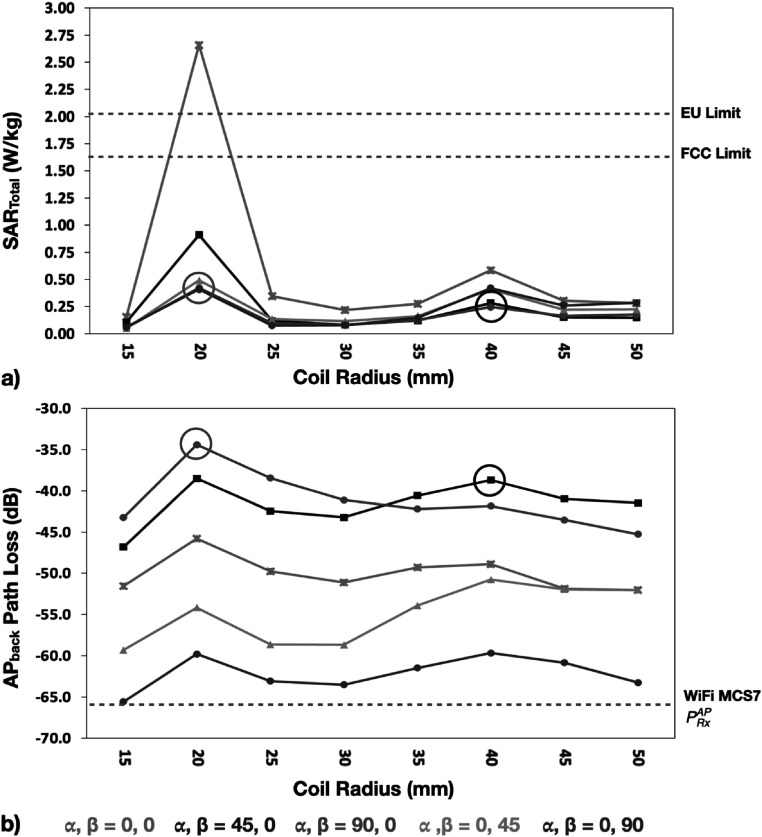
The optimal iRFW coil provided a low SAR_total_ ((a), black circle) and the second highest path loss between the iRFW coil and the AP_back_ ((b), black circle). The (*a* = 20 mm, *α* = 90°, *β* = 0°) coil radius–position combination provided a slightly improved path loss but a 48.6% increase in SAR_total_ (grey circles). The least optimal (*α* = 0°, *β* = 90°) coil radius–position combinations had an approximate 20 dB lower path loss to the AP_back_ ((b), purple line).

The 1 g and 10 g averaged SAR_MRI_ values for the optimal iRFW coil design were 0.090 and 0.056 W kg^–1^ for the optimal coil radius–position combination (figure 6(a)), respectively, which were similar to those of the traditional RF coil with the same radius and position (figure 6(c)), and they only changed by a maximum of 1.89% for all coil radii and positions. Additionally, the optimal iRFW coil provided 1 g and 10 g averaged SAR_WiFi_ values of 0.205 and 0.112 W kg^–1^ (figure 6(b)), respectively, which provided a SAR_total_ that is well within the FCC and EU specification limits (figure 7(a)).

**Figure 8.**
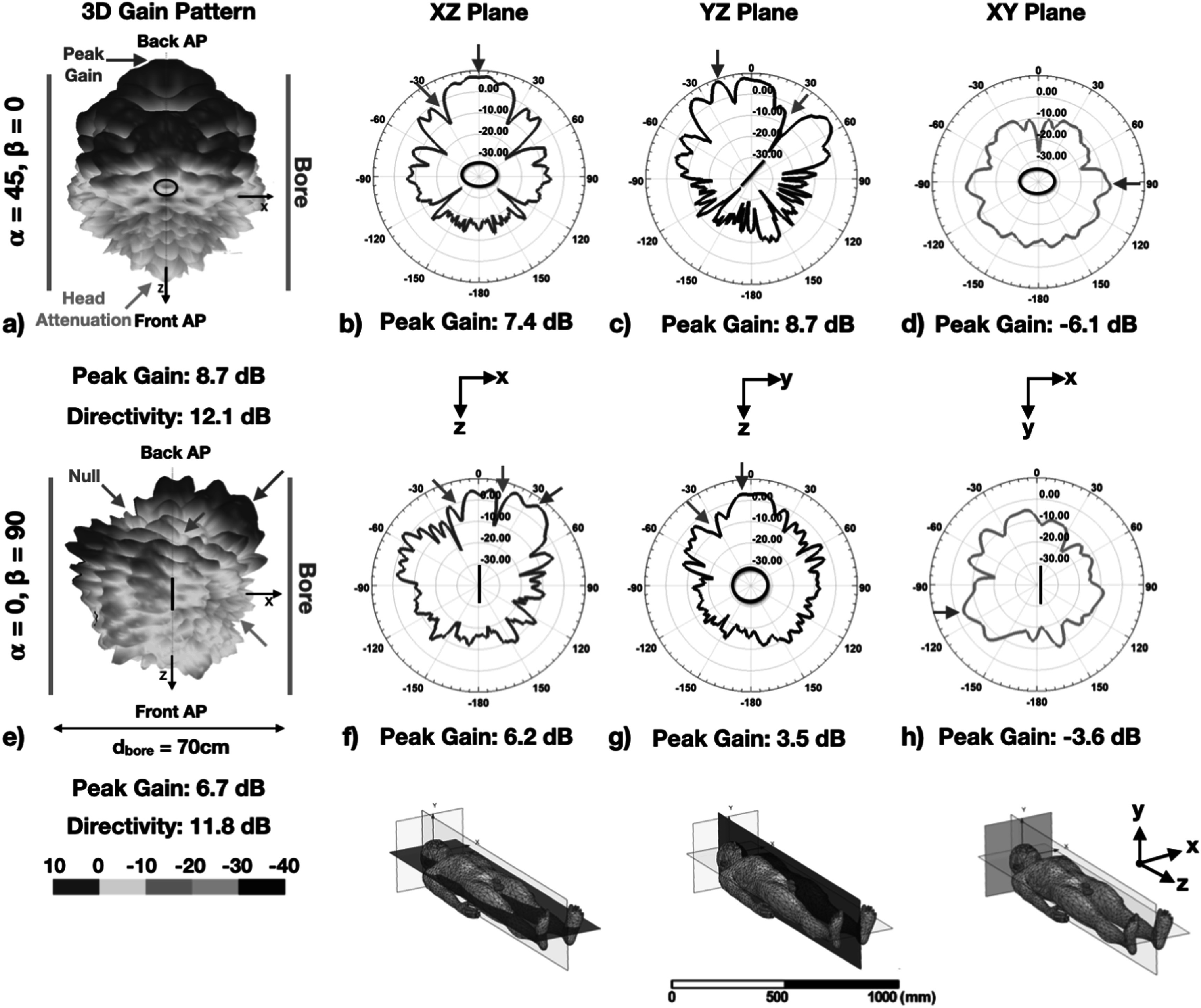
The optimal (*a* = 40 mm, *α* = 45°, *β* = 0°) iRFW coil’s 3D gain pattern had a peak gain directed along the bore axis toward the AP_back_ ((a), green arrow) and was attenuated by the human model toward the AP_front_ ((a), orange arrow). The iRFW coil’s gain in the scanner bore was predominantly in the *XZ* ((b), green arrow) and *YZ* ((c), green arrow) planes toward the AP_back_, with less gain in the cross-sectional *XY* plane of the scanner bore (d). The iRFW coil with the same radius but the least optimal (*α* = 0°, *β* = 90°) position (e) had nulls in the *XZ* ((f), magenta arrows) and *YZ* ((g), magenta arrow) planes toward the AP_back_ and an increased gain in the *XY* plane (h).

The optimal iRFW coil’s gain pattern had a peak gain of 8.7 dB and a peak directivity of 12.1 dB along the axis of the scanner bore, which allowed the majority of the radiated power to propagate along the bore axis to the AP_back_ (figure 8(a), green arrow). The peak gain values from the coil in the *XZ* (figure 8(b), green arrow) and *YZ* (figure 8(c), green arrow) principal planes were 7.4 and 8.7 dB, respectively, which were approximately along the bore axis and significantly higher than the peak gain value in the *XY* plane of –6.1 dB directed into the scanner bore (figure 8(d), green arrow). In contrast, the gain pattern for the iRFW coil with the same radius (*a* = 40 mm) but at the least optimal (*α* = 0°, *β* = 90°) position had a lower peak gain value of 6.7 dB, which was in the direction of the scanner bore (figure 8(e)). Additionally, the gain pattern had an increased number of nulls toward the AP_back_ compared to the optimal iRFW coil design. For example, the *XZ* (figure 8(f), magenta arrows) and *YZ* (figure 8(g), magenta arrow) principal planes had nulls with a value lower than –10 dB toward the AP_back_ and an increased gain in the *XY* plane of the scanner bore (figure 8(h)), which decreased the path loss.

The optimal coil radius–position combination (*a* = 40 mm, *α* = 45°, *β* = 0°) (figure 7, black circles) for these simulations had the second best path loss value to the AP_back_ of −38.7 dB and a 1 g averaged SAR_total_ of only 0.294 W kg^–1^. This combination also had a path loss to the AP_front_ of −63.0 dB, which was 13.4 dB higher than for the worst combination (*a* = 30 mm, *α* = 0°, *β* = 45°) and only 5.8 dB lower than for the best combination (*a* = 40 mm, *α* = 0°, *β* = 0°). While the (*a* = 20 mm, *α* = 90°, *β* = 0°) combination had a slightly improved path loss to the AP_back_ of −34.4 dB, it also had a 48.6% higher 1 g averaged SAR_total_ of 0.42 W kg^–1^ (figure 7, grey circles), which made it a less optimal combination. In contrast, the (*α* = 0°, *β* = 90°) position was the least optimal solution for all coil radii because the path loss to the AP_back_ (figure 7(b), purple line) was on average 20.6 dB worse than that of the optimal combination (figure 7(b), black circle) due to interactions with the scanner bore and the human model (figure 8(e)).

Finally, the simulations with the optimal iRFW coil radius–position combination for the nine human model heights between 162 and 194 cm showed a range in path loss from the optimal coil to the AP_front_ (figure 10(a), blue line) and AP_back_ (figure 10(a), red line) of only 5.1 and 1.1 dB, respectively.

### 4.2. Experimental measurements

**Figure 9.**
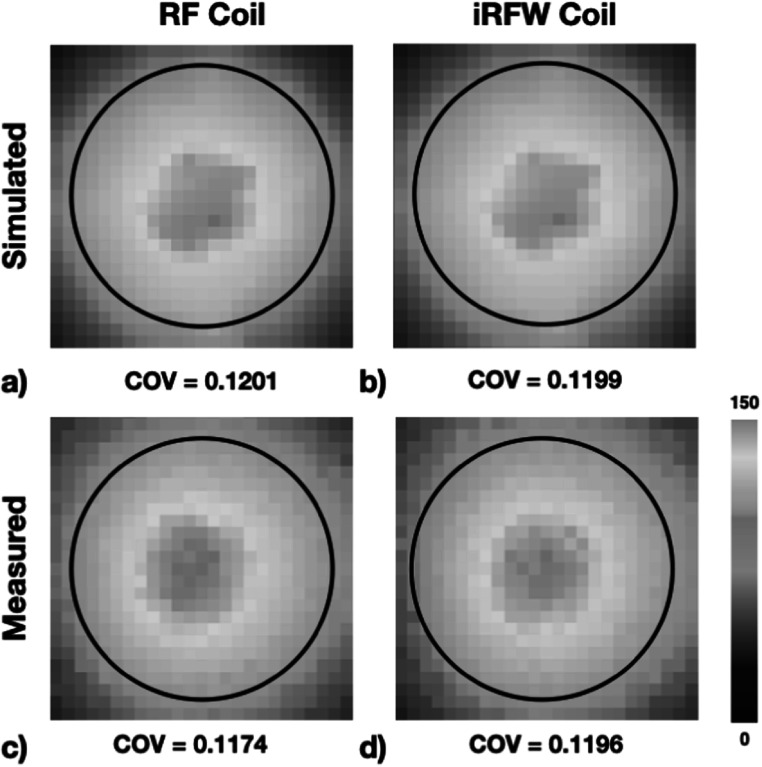
The simulated SNR maps for the traditional RF (a) and iRFW (b) coils and the corresponding measured SNR maps for the traditional RF (c) and iRFW (d) coils had similar distributions and COVs within the coil perimeter and a maximum difference in COV of 2.24% between any of the simulated or measured SNR maps.

The simulated (figures 9(a), (b)) and measured (figures 9(c), (d)) SNR COVs within the coil perimeter of the traditional RF coil and the iRFW coil all had similar spatial distributions with a maximum difference in COV of 2.24% between any of the measured or simulated SNR maps. Further, the average and maximum measured SNR values for the traditional RF coil (figure 9(c)) of 78.1 and 96.2 were in good agreement with the measured average and maximum SNR values for the iRFW coil (figure 9(d)) of 77.3 and 95.6, which only showed a difference of 0.70% and 1.07% between them. While the circuits in the simulations have been updated to have electrical characteristics like those used in the coil there remains uncertainly from the distributed inductances, capacitances and additional DCR in the construction of the filter and matching circuits that contribute to the small difference in COVs.

**Figure 10.**
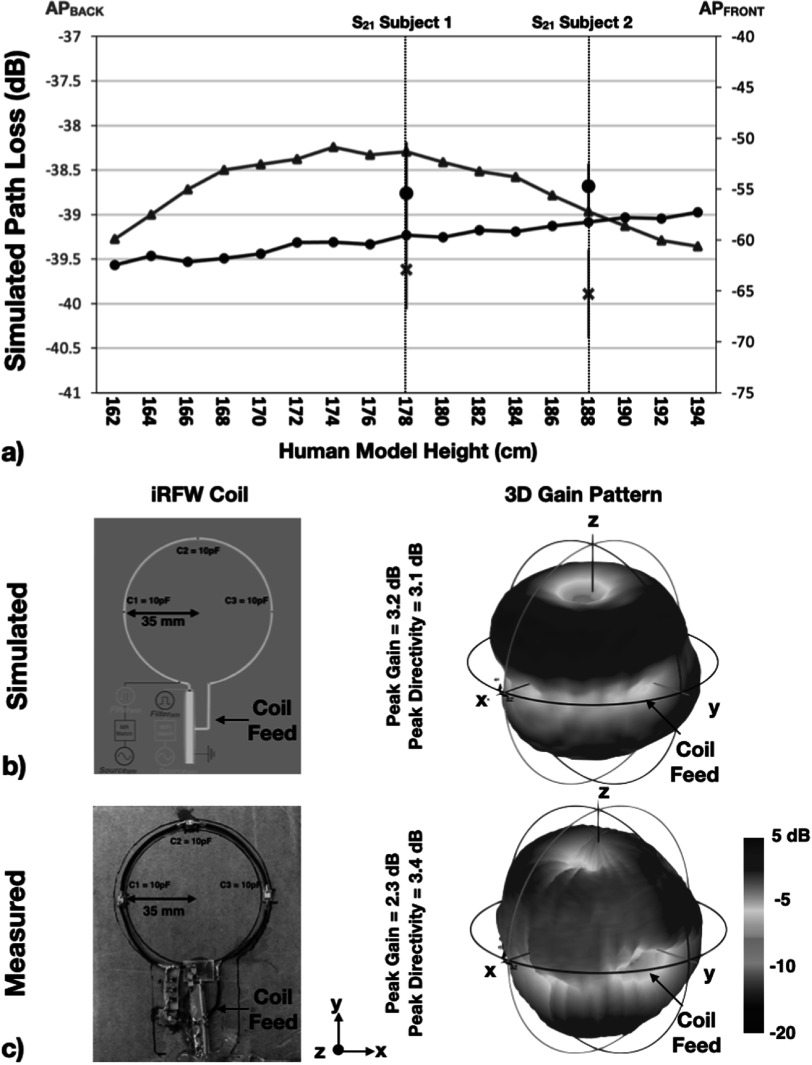
The simulated path loss between the optimal iRFW coil and the AP_front_ ((a), blue line) and AP_back_ ((a), red line) varied with the body height by only 5.1 and 1.1 dB, respectively. The average *S*_21_ isolation measurements for the optimal coil position (*α* = 45°, *β* = 0°) ((a), blue circles ± 1 standard deviation) were within 1.5 standard deviations of the simulated results. The least optimal coil position (*α* = 0°, *β* = 90°) for the coil of the same radius ((a), purple x ± 1 standard deviation) showed up to a 10 dB difference in isolation compared to the optimal coil. The simulated (b) and measured (c) free-space gain patterns for this coil were in good agreement.

The simulated and measured free-space gain patterns of the iRFW coil had similar peak directivities of 3.1 and 3.4 dB and peak gain values of 3.2 and 2.3 dB, respectively. Further, the gain patterns have the expected nulls along the normal of the coil but an additional decrease in gain in the *XY* plane from the implementation of the feed connection to the coil found in both the simulated and measured results (figures 10(b), (c), black arrow). The agreement between these simulations and measurements provides confidence in the remaining simulated gain patterns for the different coil radius–position combinations.

The average *S*_21_ isolation measurement between the optimal iRFW coil (*a* = 40 mm, *α* = 45°, *β* = 0°) placed in the scanner bore and the AP_front_ with no subject was −45.3 ± 2.9 dB, which is in strong agreement with the simulated value of −46.4 dB. The same isolation measurements with the two 178 and 186 cm tall subjects placed between the coil and the AP_front_ were −55.4 ± 4.5 and −54.7 ± 2.2 dB (figure 10(a), blue circles), which correspond to simulated path losses to the AP_front_ of −59.5 and −58.2, respectively, for human models of the same heights. Finally, the isolation measurements for the same coil placed at the least optimal position of (*α* = 0°, *β* = 90°) were −62.9 ± 3.9 and −65.3 ± 4.3 dB (figure 10(a), purple x), which correspond to simulated path losses of −68.1 and −73.8 dB, respectively.

## 5. Discussion

The simulations and experimental measurements performed in this work provide a methodology to optimize the new iRFW coil design to simultaneously acquire MR images and wirelessly transmit the data outside of the scanner bore at a high data rate. The results show that the optimal iRFW coil design has virtually the same SNR and SAR_MRI_ as those of a traditional RF coil with the same radius and position, as well as a SAR_total_ that is well within regulatory limits.

The linearly polarized gain pattern from the iRFW coil changes with both the coil radius and its position in the scanner bore because of attenuation and/or reflections by the subject and scanner. For the five simulated positions, which are also common positions to place coil elements in MRI head coil arrays, the vector components of the gain pattern polarization will be aligned with one or more of the scanner bore Cartesian axes. Since the AP gain pattern was linearly polarized in the *XZ* plane for our measurements, the iRFW coil’s gain patterns that are polarized in the same plane would be expected to have the best path loss. Based on this, it might be assumed that the iRFW coil placed at the (*α* = 90°, *β* = 0°) position would provide the best path loss because its free-space gain pattern is polarized in the *YZ* plane and because the peak gain is directed along the coil’s normal direction. However, the simulations show an improved path loss to AP_back_ of 3.2 dB with the coil at the (*α* = 45°, *β* = 0°) position inside the scanner bore (figure 8(c)) because the interactions with the scanner bore and subject change the propagation of the radiated signal from the coil to the AP_front_ and therefore how much power is coupled between them. The optimal coil design among the 40 coil radius–position combinations simulated in this work can be further refined by performing additional simulations with intermediate coil radii and/or positions between those that have been identified as having the best trade-off between path loss and SAR_WiFi_.

The SAR_total_ for the optimal coil radius combination presented in this work is well within regulatory limits but the SAR_WiFi_ contribution can be further lowered in practice by reducing the transmit power delivered from the WiFi module to the iRFW coil, which reduces the amount of radiated power absorbed by the subject at the expense of lowering the link budget to the APs by the same amount. Alternatively, a coil radius–position combination can be chosen that is further from the head model to reduce the SAR_WiFi_ but with a worse path loss (figure 7(a), *a* = 40 mm, *α* = 90°, *β* = 0°) that results in a lower wireless link budget.

The data rate required to reliably and wirelessly transfer image data acquired with an RF coil array and sampled with a 16-bit *I*/*Q* analog-to-digital converter has been estimated to be 8 Mbps/coil element (Nohava *et al* 2020, Darnell *et al* 2022), which implies that the data rate required for a 16-channel clinical coil array is ∼128 Mbps. Fortunately, off-the-shelf WiFi modules are readily available with modulation schemes that can support data rates of up to 150 Mbps. For example, a typical 2.4 GHz WiFi module transceiver (Texas Instruments, part number: WL18x7MOD) using the 2.4 GHz WiFi MCS7 MM 4K 40 MHz modulation scheme with a receive sensitivity of ${P}_{\mathrm{Rx}}^{\mathrm{AP}}$ = −66.3 dBm can transmit up to 150 Mbps. Here, the line-of-sight link budget for the 182 cm human model placed between the AP_back_, with an ideal dipole peak gain of ${G}_{\mathrm{AP}}^{\mathrm{peak}}$ = 2.2 dBi, and the optimal iRFW coil, with a ${G}_{\mathrm{coil}}^{\mathrm{peak}}$ = 8.7 dB, corresponding to PL = 38.7 dB and transmitting at a maximum power of ${P}_{\mathrm{Tx}}^{\mathrm{coil}}$ = 12.6 dBm from the data sheet, has a large value of 51.1 dB. This large link budget allows the AP to be placed up to 6 m instead of 3 m away from the scanner isocenter because the additional 3 m free-space signal path loss at this frequency of –49.7 dB still provides a link budget of 1.4 dB for wireless data transfer, which is beneficial for larger MRI scanner rooms. Further, the path loss to the AP_back_ only varies by ∼1.1 dB for all the simulated body heights (figure 10(a), red line), which shows that the link budget would be sufficient for a range of patients. In contrast, the iRFW coil placed at the least optimal (*α* = 0°, *β* = 90°) position with a path loss to the AP_back_ of PL = −59.7 dB, as shown by the simulations would decrease the link budget by more than 20 dB, which restricts AP_back_ placement in the scanner room. Alternatively, the mm-wave wireless transfer design can achieve a Gbps data rate but requires significant modifications to the scanner (Aggarwal *et al* 2017).

The wireless link budget, and thus the data rate, between an iRFW coil element in a traditional RF coil array and an AP would minimally decrease if a F_WiFi_ is inserted between each of the traditional RF coil elements and their preamps. Specifically, each traditional RF coil element in the array would be made to include the same *F*_WiFi_ as the iRFW coil element MRI port, which provides more than –25 dB of isolation between the traditional RF and the iRFW coil elements. For this filter, approximately 3 mW of the original 1 W of RF-power delivered from the WiFi port to the iRFW coil element would be coupled to each of the 15 traditional RF coil elements in, for example, a 16-channel coil array. This corresponds to a total radiated power loss of the iRFW coil element in the WiFi frequency band to the remaining 15 traditional RF coil elements in the array of 45 mW (i.e. 3 mW × 15 traditional coil elements), which results in a 0.2 dB decrease in the wireless link budget. Further, since the *F*_WiFi_ insertion loss is less than 0.1 dB the integration of the iRFW coil element will have minimal impact on the SNR (figure 9).

The path loss between the iRFW coil and the AP_front_ for all coil radii and positions is not sufficient for wireless transfer of MRI data at an HDR, but is appropriate to wirelessly transmit data from, for example, peripheral devices at an LDR for MRI applications. Using the optimal coil radius–position combination with a path loss to the AP_front_ of −63.0 dB and the same WiFi module operating at a 2.4 GHz DSSS 1 Mbps modulation scheme with a receive sensitivity of ${P}_{\mathrm{Rx}}^{\mathrm{AP}}$ = −95.0 dBm and the specified maximum transmit power of ${P}_{\mathrm{Tx}}^{\mathrm{coil}}$ = 17.3 dBm provides a link budget of 31.2 dB. For LDR applications, this large link budget allows the module to operate at a lower transmit power and still maintain the wireless data quality, which would decrease the power consumption from battery-operated iRFW coil systems. Interestingly, these simulations show that the iRFW coil can have multiple wireless data streams to the AP_front_ and AP_back_ for different data rates, which opens new wireless applications in MRI.

## Data Availability

The data cannot be made publicly available upon publication because the cost of preparing, depositing and hosting the data would be prohibitive within the terms of this research project. The data that support the findings of this study are available upon reasonable request from the authors.
